# PgFur participates differentially in expression of virulence factors in more virulent A7436 and less virulent ATCC 33277 *Porphyromonas gingivalis* strains

**DOI:** 10.1186/s12866-019-1511-x

**Published:** 2019-06-11

**Authors:** Michał Śmiga, Paulina Stępień, Mariusz Olczak, Teresa Olczak

**Affiliations:** Laboratory of Medical Biology, Faculty of Biotechnology, University of Wrocław, F. Joliot-Curie 14A St, 50-383 Wrocław, Poland

**Keywords:** *Porphyromonas gingivalis*, Ferric uptake regulator, PgFur, Iron, Heme, Virulence

## Abstract

**Background:**

*Porphyromonas gingivalis* is considered a keystone pathogen responsible for chronic periodontitis. Although several virulence factors produced by this bacterium are quite well characterized, very little is known about regulatory mechanisms that allow different strains of *P. gingivalis* to efficiently survive in the hostile environment of the oral cavity, a typical habitat characterized by low iron and heme concentrations. The aim of this study was to characterize *P. gingivalis* Fur homolog (PgFur) in terms of its role in production of virulence factors in more (A7436) and less (ATCC 33277) virulent strains.

**Results:**

Expression of a *pgfur* depends on the growth phase and iron/heme concentration. To better understand the role played by the PgFur protein in *P. gingivalis* virulence under low- and high-iron/heme conditions, a *pgfur*-deficient ATCC 33277 strain (TO16) was constructed and its phenotype compared with that of a *pgfur* A7436-derived mutant strain (TO6). In contrast to the TO6 strain, the TO16 strain did not differ in the growth rate and hemolytic activity compared with the ATCC 33277 strain. However, both mutant strains were more sensitive to oxidative stress and they demonstrated changes in the production of lysine- (Kgp) and arginine-specific (Rgp) gingipains. In contrast to the wild-type strains, TO6 and TO16 mutant strains produced larger amounts of HmuY protein under high iron/heme conditions. We also demonstrated differences in production of glycoconjugates between the A7436 and ATCC 33277 strains and we found evidence that PgFur protein might regulate glycosylation process. Moreover, we revealed that PgFur protein plays a role in interactions with other periodontopathogens and is important for *P. gingivalis* infection of THP-1-derived macrophages and survival inside the cells. Deletion of the *pgfur* gene influences expression of many transcription factors, including two not yet characterized transcription factors from the Crp/Fnr family. We also observed lower expression of the CRISPR/Cas genes.

**Conclusions:**

We show here for the first time that inactivation of the *pgfur* gene exerts a different influence on the phenotype of the A7436 and ATCC 33277 strains. Our findings further support the hypothesis that PgFur regulates expression of genes encoding surface virulence factors and/or genes involved in their maturation.

**Electronic supplementary material:**

The online version of this article (10.1186/s12866-019-1511-x) contains supplementary material, which is available to authorized users.

## Background

Periodontal diseases, the most common chronic infections in humans, are characterized by the loss of tooth-supporting tissues [1, 2]. Analyses of subgingival samples revealed the presence and relative abundance of periodontal pathogens, including the “red complex” bacteria (*Porphyromonas gingivalis*, *Tannerella forsythia* and *Treponema denticola*) [3], which are associated with the clinical features of chronic periodontitis [4, 5]. Other bacteria, such as *Streptococcus gordonii*, a member of the “yellow complex”, and *Prevotella intermedia*, a member of the “orange complex”, serve as early colonizers or bridging species to members of the “red complex” [3, 6, 7]. Among the putative periodontopathogens, *P. gingivalis* is considered the main etiologic agent and a keystone pathogen responsible for initiation and progression of chronic periodontitis [8, 9]. *P. gingivalis* is a constituent of a mixed-species biofilm of the oral cavity [10, 11]; however, it can also enter gingival epithelial and immune cells, and can spread among host cells, thus contributing to its survival in the oral cavity [12–16].

*P. gingivalis* is a heme auxotroph and uptake of this compound is required as an iron and protoporphyrin IX source, as well as being crucial for survival and efficient establishment of infection by this bacterium. Among well-characterized heme acquisition systems of *P. gingivalis* is that encoded by the *hmu* operon, comprising HmuR, a typical TonB-dependent receptor involved in heme transport through the outer membrane, HmuY, a heme-binding hemophore-like protein exhibiting unique structure and heme-binding properties, and four proteins with, as yet, unknown function [17]. *P. gingivalis* produces higher levels of HmuY when the bacterium grows under low-iron/heme conditions or as a biofilm constituent [18, 19], as well as intracellularly in host cells [16]. The HmuY protein is associated with both the bacterial outer membrane and outer-membrane vesicles (OMVs) through a lipid anchor [19, 20], and can also be shed as an intact, soluble protein as a result of the limited proteolytic processing by *P. gingivalis* lysine-specific gingipain K (Kgp) [20, 21]. Heme can also be acquired from host hemoproteins through the high proteolytic activity of *P. gingivalis*, caused mainly by a group of cysteine proteases, comprising secreted and membrane-associated gingipains [22, 23]. Arginine-specific gingipains (RgpA and RgpB) are encoded by the *rgpA* and *rgpB* genes, and Kgp by the *kgp* gene. The predominant form of Kgp and RgpA is a complex of a catalytic domain with hemagglutinin/adhesion domains.

Although several mechanisms of *P. gingivalis* virulence, including uptake of iron, heme, and other nutrients are quite well characterized [17, 23], very little is known about regulatory mechanisms that allow *P. gingivalis* to survive in the very hostile environment of the oral cavity, a typical habitat characterized by low iron and heme availability. Regulation of gene expression in bacteria is usually carried out at the transcriptional level by DNA-binding proteins and at the post-transcriptional level by protein-protein interactions [24]. Among the best characterized bacterial regulators, there is a ferric uptake regulator (Fur), a member of the FUR superfamily, comprising a variety of metalloregulators, which can regulate expression of genes at the transcriptional level in response to environmental factors through several different mechanisms [25, 26]. In the classical Fur regulation paradigm, under high-iron conditions Fur becomes ferreted and binds to specific “Fur box” sequences located in promoters of regulated genes, thereby preventing their transcription through steric hindrance of RNA polymerase. Under iron-restricted conditions, the Fur-Fe^2+^ complex dissociates and the repressor becomes inactive, which leads to the transcription of target genes. In contrast to iron-dependent DNA binding activity, Fur in some systems can sense signals other than iron, including heme, or bind to DNA in the absence of a metal cofactor [27–29]. Importantly, it has been well documented that Fur functions as a global regulator that controls not only the expression of iron acquisition systems, but also a large number of genes involved in different cellular processes, such as stress response and production of virulence factors [25, 26, 30].

*P. gingivalis* possesses a single predicted Fur homolog, which we termed PgFur [31]. Our recent data suggested that iron and heme limitation together with inactivation of the *pgfur* gene affects directly or indirectly the expression of several genes encoding important virulence factors of *P. gingivalis* A7436 strain. According to our hypothesis, *P. gingivalis* employs PgFur to initiate a complex, multilayer network used to regulate virulence in the host iron/heme-limited environment. To date, several genomes of *P. gingivalis* strains obtained from patients have been sequenced and deposited in databases. These strains differ in both the genotype and the exhibited phenotype. Therefore, the aim of this study was to characterize the importance of *P. gingivalis* PgFur in the production of virulence factors in more (A7436) and less (ATCC 33277) virulent strains.

## Methods

### Bacterial strains and growth conditions

*P. gingivalis* wild-type (A7436, ATCC 33277), *pgfur* mutant (TO6, TO16), complemented mutant (TO6 + *pgfur*, TO16 *+ pgfur*), and control (TO6 + pTIO-tetQ, TO16 + pTIO-tetQ) strains were grown anaerobically at 37 °C for 3–10 days on blood agar plates (ABA) (Biocorp) as described previously [18, 31]. These cultures were used as the inoculum for growth in liquid basal medium (BM) comprising 3% trypticase soy broth (Biocorp), containing 0.5% yeast extract (Biocorp), 0.05% cysteine (Sigma), 0.5 mg/l menadione (Fluka), and supplemented with 7.7 μM hemin (Hm) or 0.1–1.0 mM ZnCl_2_ (Zn). To mimic low-iron/heme conditions, bacteria were grown in BM in the absence of heme and in the presence of an iron chelator, 160 μM 2,2-dipyridyl (DIP) (Sigma) or 0.5 mM EDTA (EDTA) (Sigma). To examine the influence of oxidative stress, bacteria were grown in BM lacking cysteine and supplemented with 0.05 or 0.25 mM H_2_O_2_. TO6 and TO16 strains were maintained in the presence of 1 μg/ml erythromycin, whereas complemented mutant (TO6 + *pgfur*, TO16 *+ pgfur*) and control (TO6 + pTIO-tetQ, TO16 + pTIO-tetQ) strains in the presence of 1 μg/ml erythromycin and 1 μg/ml tetracycline. Bacterial growth in liquid culture media was determined by measuring optical density at 600 nm (OD_600_) and on ABA plates by visual inspection.

### Construction of *P. gingivalis pgfur* mutant, complemented mutant and control strains

Inactivation of the *P. gingivalis pgfur* gene (*PGA7_00014570*) in the A7436 wild-type strain was previously reported [31], resulting in the TO6 mutant strain. The *P. gingivalis pgfur* gene in the wild-type ATCC 33277 strain (*PGN_1503*) was analogously inactivated in this study by introducing the *ermF* cassette (encoding the erythromycin resistance gene from *Bacteroides fragilis*) to yield a *pgfur-ermF-pgfur* DNA fragment, resulting in the TO16 mutant strain. In addition, complemented mutant (TO6 + *pgfur*, TO16 *+ pgfur*) and control (TO6 + pTIO-tetQ, TO16 + pTIO-tetQ) strains were constructed using the modified pTIO-1 vector, originally designed and constructed by Tagawa et al. [32], for electroporation of *P. gingivalis*. Briefly, the *ermF* cassette in the original pTIO-1 plasmid was first replaced by the *tetQ* cassette from *B. thetaiotaomicron * (encoding the tetracycline resistance gene), yielding the pTIO-tetQ plasmid. This modified plasmid was introduced into TO6 and TO16 mutant strains to construct control TO6 + pTIO-tetQ and TO16 + pTIO-tetQ strains. To construct complemented mutant strains, the *pgfur* gene with the original promoter region was introduced into XhoI and BamHI restriction sites of the pTIO-tetQ plasmid. All primers used in this study are listed in Additional file 1: Table S1. *P. gingivalis* strains were electroporated with appropriate plasmids and subsequently grown as described previously [31, 32]. All constructs and homologous recombinations between the constructs and the chromosomal DNA of *P. gingivalis* were verified by PCR and sequencing analyses (data not shown).

### Co-aggregation assay

*P. gingivalis*, *T. forsythia* ATCC 43037 and *P. intermedia* 17 were grown under anaerobic conditions as described previously [33]. *Streptococcus gordonii* ATCC 10558 was grown in 5% Tryptic soy broth (TSB, Becton Dickinson) under a CO_2_-enriched atmosphere (Thermo Scientific). To examine the co-aggregation ability of *P. gingivalis* wild-type and *pgfur* mutant strains with other species, bacteria were incubated anaerobically for 6 h in 20 mM phosphate buffer, pH 7.4, containing 140 mM NaCl (PBS). The co-aggregation ability was determined by measuring the decrease in OD at 600 nm.

### Microarray analysis

Genome-wide expression profiling was carried out (IMGM, Martinsried, Germany) using *P. gingivalis* wild-type (A7436 and ATCC 33277) and *pgfur* mutant (TO6 and TO16) strains, grown under low- (DIP) or high-iron/heme (Hm) conditions analogously as described previously for *P. gingivalis* A7436 wild-type and TO6 mutant strains [31].

### Quantitative reverse transcriptase-polymerase chain reaction (qRT-PCR)

RNA was extracted from 0.5 × 10^8^–4 × 10^8^ *P. gingivalis* cells using the Total RNA Mini Kit (A&A Biotechnology). Isolated RNA was treated with DNase I (A&A Biotechnology) and purified using the Clean-Up RNA Concentrator Kit (A&A Biotechnology). RNA integrity was verified using a spectrophotometric method and separating RNA samples on an agarose gel. Reverse transcription was carried out using 1 μg of RNA and the SensiFAST cDNA Synthesis Kit (Bioline).

PCR was carried out using the SensiFAST SYBR No-ROX Kit (Bioline) and the LightCycler 96 System (Roche). The amplification reaction comprised initial denaturation at 95 °C for 2 min, 40 cycles of denaturation at 95 °C for 5 s, primer annealing at 60 °C for 10 s, and extension at 72 °C for 20 s. The melting curves were analyzed to monitor the quality of PCR products. Relative quantification of expression of respective genes was determined in comparison with the *16S rRNA* gene of *P. gingivalis* (*PGA7_00000960* for A7436 and *PGN_r0001* for ATCC 33277) as a reference, using the Pfaffl method [34]. All samples were run in triplicate for the target and reference genes, in three independent experiments. No-template controls were included on each reaction plate to check for contamination. Negative controls consisting of untranscribed RNA (no-RT controls) were performed to check for genomic DNA contamination. All primers used in this study are listed in Additional file 1: Table S1.

### Sodium dodecyl sulfate-polyacrylamide gel electrophoresis (SDS-PAGE) and Western blotting

Proteins were prepared by sample incubation with Laemmli loading buffer (95 °C, 10 min, 1200 rpm) and separated in 12% polyacrylamide gels using SDS-PAGE as described previously [31]. The protein amount of cell lysate loaded per well was normalized against *P. gingivalis* culture volume and the number of cells determined by measuring OD at 600 nm. Proteins were either visualized by Coomassie Brilliant Blue G-250 (CBB G-250) staining or transferred onto nitrocellulose membranes (Millipore). HmuY protein was detected with anti-HmuY antibodies as described previously [33].

### Lectin assay

An equivalent of OD_600_ = 0.3 of bacterial cultures was separated using SDS-PAGE and transferred onto nitrocellulose membranes. After blocking with Carbo-Free Blocking Solution (Vector Laboratories), membranes were incubated with biotinylated lectins (1:3300; Vector Laboratories) in 50 mM Tris–HCl, pH 7.5, containing 150 mM NaCl (TBS), 0.2% Tween-20, 1 mM CaCl_2_ and 1 mM MnCl_2_. Lectin binding was detected using alkaline phosphatase-conjugated avidin D (Vector Laboratories) and visualized with NBT/BCIP substrate solution (Roche). Equal protein loading was determined by staining proteins in gels using CBB G-250 or on a nitrocellulose membrane by staining with Ponceau S.

### Bacterial cell fractionation

Bacterial cultures were centrifuged at 20,000×*g* for 20 min at 4 °C. Bacterial pellets were washed twice with PBS and used to analyze the whole cell fraction (WC). The supernatant was filtered using 0.22 μm filters to obtain the cell-free fraction and subsequently centrifuged at 100,000×*g* for 2 h at 4 °C to separate the outer-membrane vesicles (OMV) fraction. The soluble fraction (cell-free supernatant; CM) was concentrated 25× using Amicon Ultra-4 Centrifugal Filter Ultracel-10 K units (Millipore).

### Determination of proteolytic activity

Whole *P. gingivalis* cultures or cell-free culture media were examined in order to determine proteolytic activity. The total proteolytic activity was measured using azocasein protease substrate according to the manufacturer’s protocol (Sigma). To analyze proteolytic activity specific for Arg- and Lys-specific gingipains, Nα-benzoyl-DL-arginine 4-nitroanilide hydrochloride (BApNA) (Sigma) and N-(p-tosyl)-Gly-Pro-Lys 4-nitroanilide acetate salt (Sigma) were used, respectively [35].

### THP-1 cultures and infection assay

Human acute monocytic leukemia cells (THP-1) were grown in RPMI-1640 medium (Biowest), supplemented with 10% heat-inactivated fetal bovine serum (FBS, Cytogen), 2 mM L-glutamine (Cytogen), 100 U/ml penicillin and 100 μg/ml streptomycin (Cytogen) in a humidified atmosphere of 95% air and 5% CO_2_ at 37 °C in a CO_2_ incubator (Sanlab). THP-1 cells were seeded at 1.0 × 10^6^/ml in 12-well plates and differentiated toward macrophages by treatment with 0.01 μg/ml phorbol 12-myristate 13-acetate (PMA, Sigma Aldrich) for 48 h, as described previously [36].

Live *P. gingivalis* wild-type and *pgfur* mutant strains were grown to the early stationary phase in BM + Hm, pelleted by centrifugation (4000×*g*, 20 min at 4 °C), and washed twice with PBS. Bacteria were added to THP-1-derived macrophages cultured without antibiotics in 12-well plates at a multiplicity of infection (MOI) of 100 and incubated for 4 h at 37 °C as described previously [31]. Then, the cells were washed 3 times with PBS and one portion out of four portions was lysed with water to determine bacteria attached to and present inside the host cells. To the remaining samples, fresh RPMI 1640 medium was added, supplemented with 300 μg/ml gentamicin and 200 μg/ml metronidazole. After 1 h, one cell portion was washed 3 times with PBS and lysed to determine the amount of live cells present inside the host cells. After 8 and 24 h, the two last cell portions were washed 3 times with PBS and lysed to determine live bacteria present inside the host cells. Serial dilutions of samples were plated on blood agar plates and colony forming units per ml (CFU/ml) were determined. All experiments were performed three times in duplicate.

## Results

In our previous study, we reported preliminary analysis of a *pgfur* mutant strain (TO6) constructed in the more virulent A7436 wild-type strain [31] and suggested that PgFur can influence in vivo *P. gingivalis* growth and virulence, allowing efficient infection through a complex regulatory network. To provide more accurate and deeper understanding of the biological role played by PgFur in the production of virulence factors, we inactivated the *P. gingivalis pgfur* gene also in the less virulent ATCC 33277 strain. Complementation of the mutant strains with the native *pgfur* gene resulted in partial reversion of the mutants’ phenotypes (Additional file 2: Fig. S1a and data not shown). We also demonstrated that introduction of the pTIO-tetQ plasmid alone to the mutant strains did not change control strains’ phenotypes (data not shown).

### Expression of *pgfur* gene

**Fig. 1:**
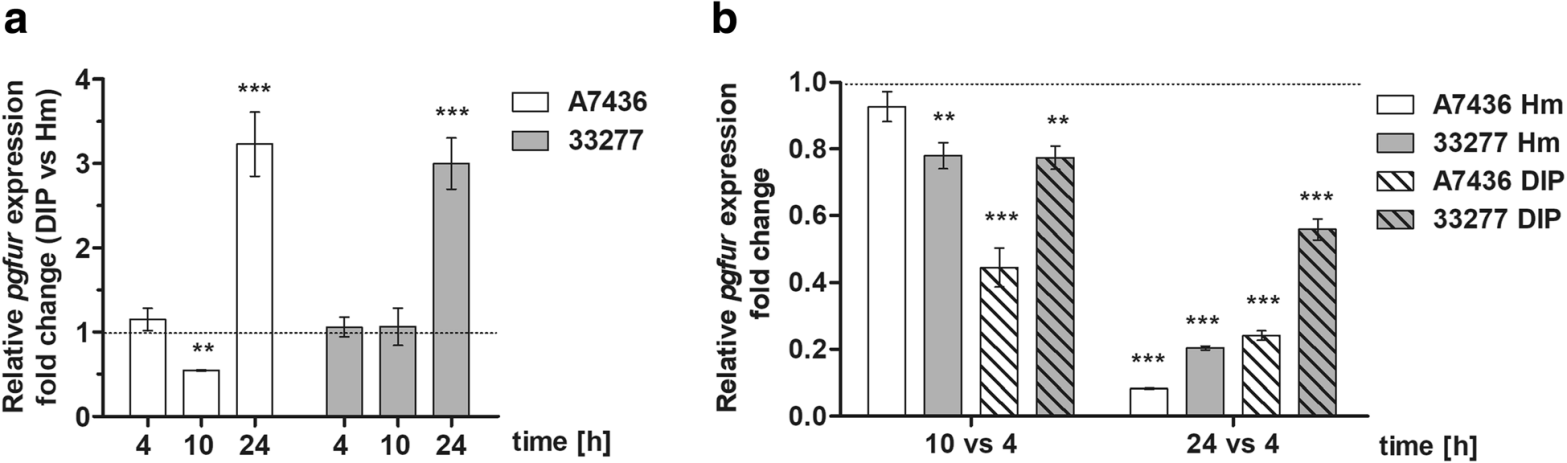
Expression of *pgfur* gene in *P. gingivalis* strains. Relative expression of the *pgfur* gene was determined using RT-qPCR and shown in relation to iron/heme availability (**a**) and additionally to growth phase (**b**) at the indicated time points. Data are shown as relative levels of the mRNA normalized to *16S rRNA* mRNA and presented as mean ± standard deviation (mean ± SD) from three replicates. **P < 0.01, ***P < 0.001

Determination of the *pgfur* transcript in both *P. gingivalis* wild-type strains showed differences when low-iron/heme (DIP) conditions were compared at the indicated time points with high-iron/heme (Hm) conditions (Fig. 1 a). In general, our results demonstrated that in both strains mRNA encoding PgFur protein is produced at higher levels after prolonged growth (24 h) when bacteria grow under low iron/heme conditions. Interestingly, the A7436 strain under these conditions exhibited unusual decreased *pgfur* gene expression after 10 h.

Further we analyzed influence of the growth phase on *pgfur* gene expression by comparing its expression determined after 10 and 24 h with the expression determined after 4 h in bacteria grown under low- or high-iron/heme conditions. The highest *pgfur* expression was observed at the early growth phase for both A7436 and ATCC 33277 strains (Fig. 1 b). This effect was more pronounced when bacteria grew under low-iron/heme conditions, and when bacterial cultures at early stationary phase were compared with the growth at the early stage of the cultures. This would suggest that culture density might influence expression of the *pgfur* gene.

### Phenotypic characterization of *pgfur* mutant strains

**Fig. 2:**
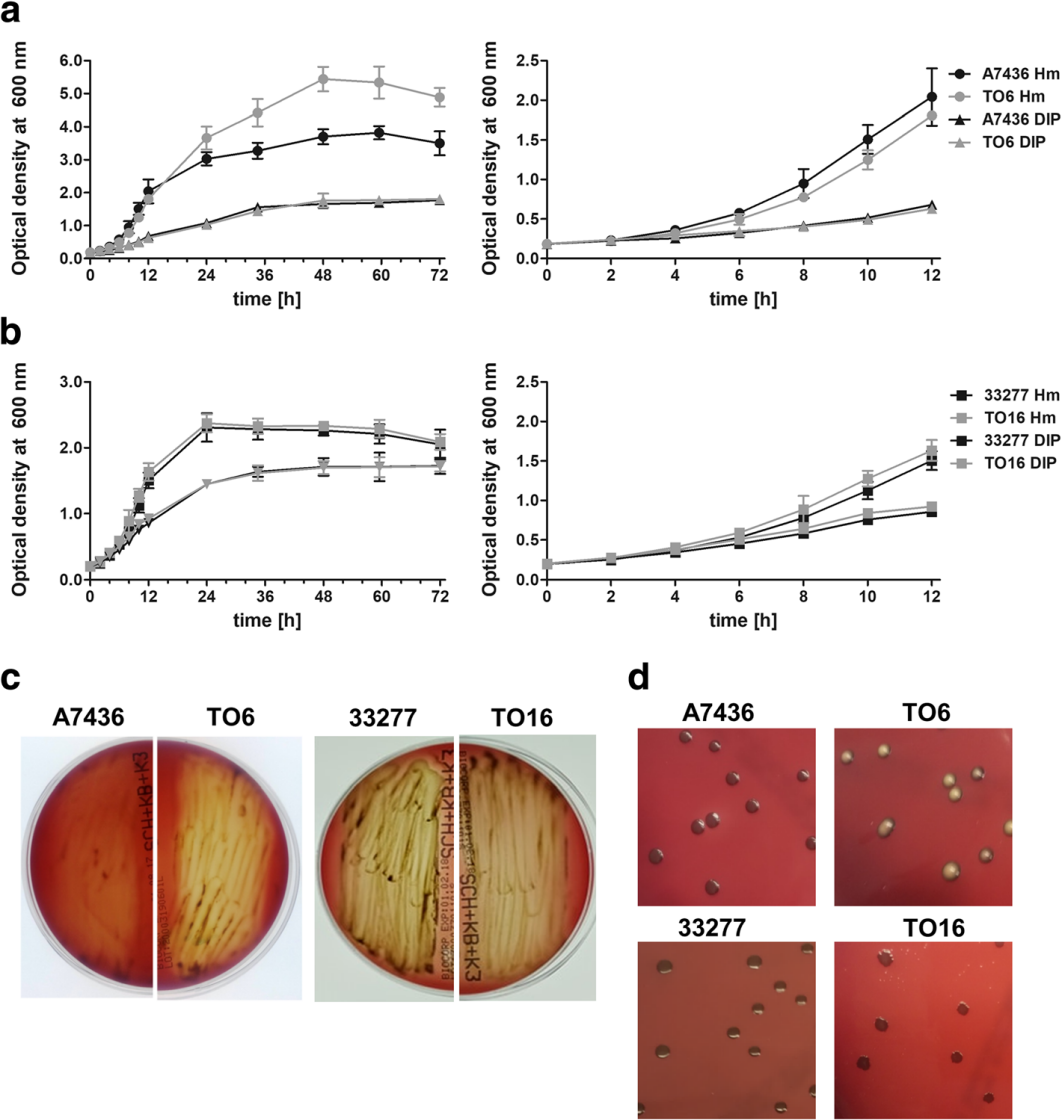
Phenotypic characterization of *P. gingivalis pgfur* mutant strains. **a**, **b** *P. gingivalis* wild-type (A7436 and ATCC 33277) and *pgfur* mutant (TO6 and TO16) strains were grown in liquid medium. Bacterial cultures were started at OD_600_ ~ 0.2 and the growth in basal medium supplemented with 7.7 μM hemin (Hm) or 160 μM dipyridyl (DIP) was monitored by measuring optical density at 600 nm at the indicated time points. **c**, **d** Growth of *P. gingivalis* wild-type (A7436 and ATCC 33277) and *pgfur* mutant (TO6 and TO16) strains on blood agar plates. Hemolysis (**c**) and colony color and shape (**d**) were visually observed for up to 10 days and pictures taken after 3 (**c**) and 5 (**d**) days, respectively are shown. Representative data out of 3 independent experiments performed in triplicate with a similar tendency are shown

Characterization of phenotypes of the *pgfur* mutant strains revealed different morphological and physiological effects of the gene inactivation in the two *P. gingivalis* strains. In accordance with our previous studies [31], growth of the TO6 mutant strain in liquid media under high-iron/heme conditions (Hm) resulted in higher cell density compared with the A7436 wild-type strain (Fig. 2 a). In contrast, the TO16 mutant strain did not differ significantly in growth ability in liquid media under these conditions compared with the ATCC 33277 wild-type strain (Fig. 2 b). These findings did not correlate with the *luxR* gene expression, one of the components responding to bacterial culture density (Additional file 3: Table S2). To produce iron/heme-depleted conditions, dipyridyl (DIP) was used and heme was not added to the basal medium. No significant differences in growth ability in the case of all examined strains were observed under these conditions (Fig. 2 a and b). To analyze whether other metals, such as zinc, may affect *P. gingivalis* growth, EDTA was used. This compound was less effective in ion chelation, resulting in better *P. gingivalis* growth compared with DIP. Also under these conditions, no significant differences were found between the strains examined (data not shown). In addition, *P. gingivalis* growth in the presence of zinc excess (Zn) was analyzed. As expected, these conditions caused growth inhibition; however, both wild-type and mutant strains were able to recover and re-start replication in the presence of up to 1 mM ZnCl_2_ (data not shown). The only difference was significantly longer time required for growth recovery of ATCC 33277 and TO16 strains (e.g., ~ 24 h in the presence of 0.5 mM ZnCl_2_) compared with A7436 and TO6 strains (e.g., ~ 16 h in the presence of 0.5 mM ZnCl_2_).

When bacteria were grown on blood agar plates for a prolonged time, a significantly higher hemolytic activity (Fig. 2 c) and brighter colony color (Fig. 2 d) of the TO6 strain were observed compared with both wild-type strains and the TO16 mutant strain. When the ability of heme binding to the cell surface of whole live bacteria was examined, no significant differences, except lower heme binding in the case of the TO6 mutant strain, were demonstrated (Additional file 4: Figure S2). This might, at least in part, explain the lower colony pigmentation of the TO6 mutant cells (Fig. 2 d). Interestingly, the mutant TO16 strain exhibited different colony shape compared with the wild-type strain (Fig. 2 d).

**Fig. 3:**
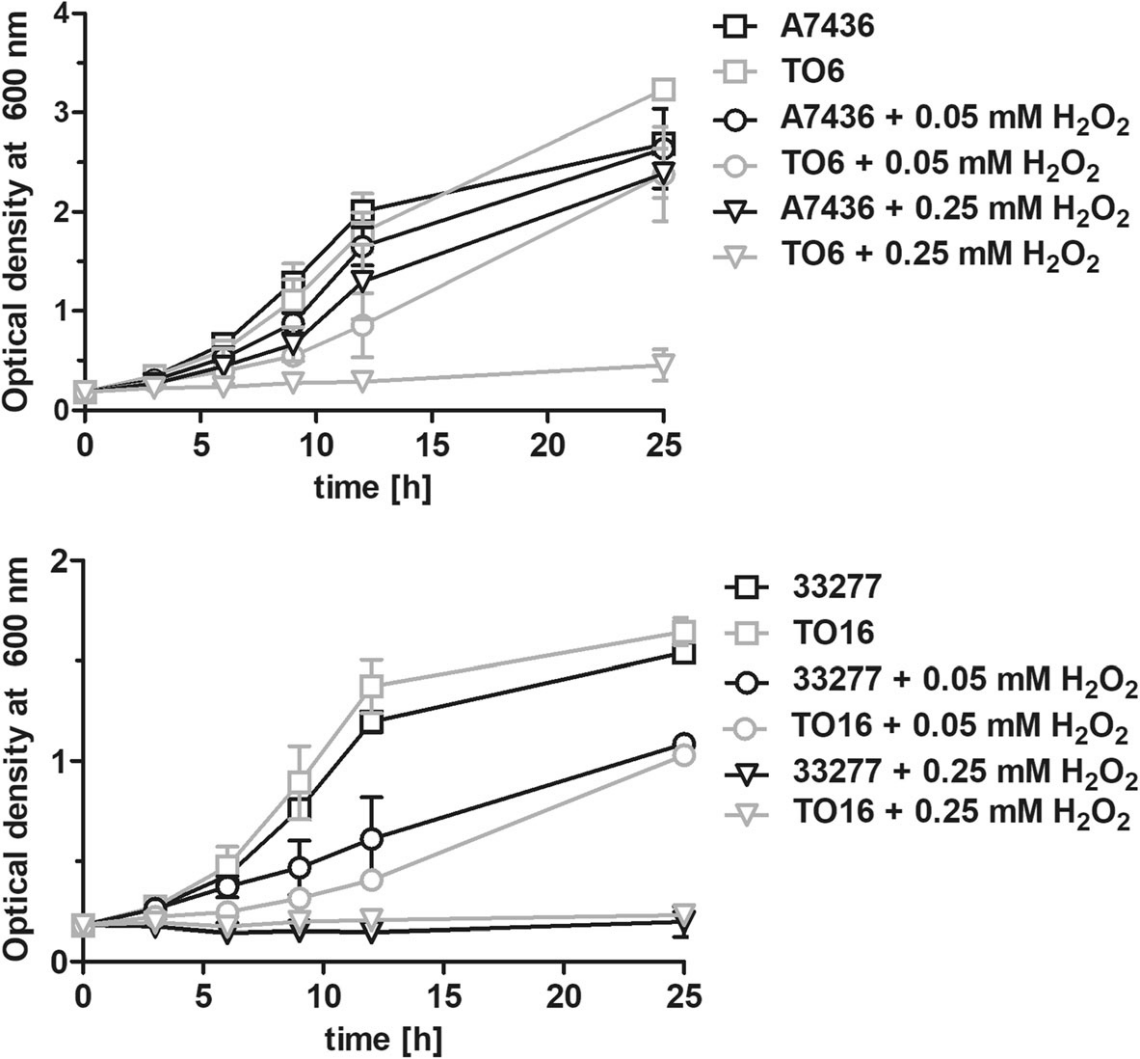
*P. gingivalis* growth in a liquid medium in the presence of H_2_O_2_. Bacterial cultures were started at OD_600_ ~ 0.2 and the growth in basal medium supplemented with 7.7 μM hemin and H_2_O_2_ was monitored by measuring optical density at 600 nm at the indicated time points

Proteins belonging to Fur family may also control the expression of genes engaged in redox sensing [37]. Therefore, we examined *pgfur* mutant strains as regards their susceptibility to oxidative stress. Data shown in Fig. 3 demonstrated that both TO6 and TO16 mutant strains were more susceptible to oxidizing conditions. In addition, the ATCC 33277 wild-type strain was more susceptible to the negative effect of H_2_O_2_ compared with the A7436 wild-type strain. However, these findings did not correlate with *oxyR* gene expression examined under different iron and heme availability (Additional file 4: Table S2).

**Fig. 4:**
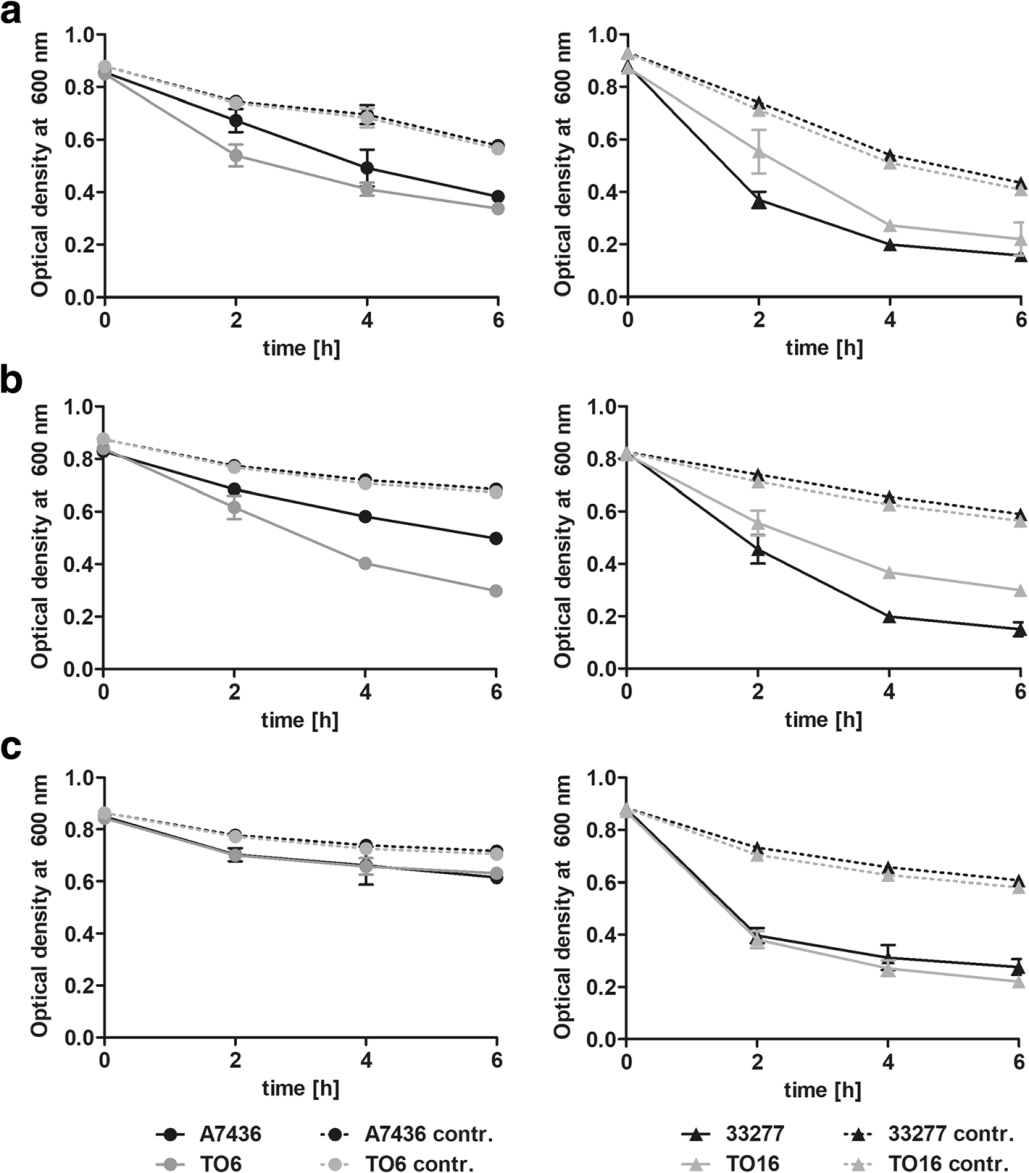
Interaction of *P. gingivalis* wild-type (A7436 and ATCC 33277) and *pgfur* mutant (TO6 and TO16) strains with *T. forsythia* (**a**), *P. intermedia* (**b**), and *S. gordonii* (**c**). The tendency to co-aggregate was monitored by measuring optical density at 600 nm at the indicated time points. Control samples contain mono-species cultures

To demonstrate the influence of the PgFur on interaction ability with other oral bacteria, co-aggregation between *P. gingivalis* and selected species was examined. As shown in Fig. 4 a and b (left panel), the TO6 mutant strain co-aggregated more efficiently with *T. forsythia* and *P. intermedia*, compared with the A7436 strain. Surprisingly, the TO16 mutant strain exhibited the opposite effect (Fig. 4 a and b; right panel). No differences between the wild-type and *pgfur* mutant strains were observed when *P. gingivalis* and *S. gordonii* were examined (Fig. 4 c).

### Gene expression after *pgfur* inactivation

To analyze expression of *P. gingivalis* genes upon *pgfur* inactivation on the global scale, we employed microarray analysis and compared the gene expression profiles between the wild-type and mutant strains grown in liquid iron and heme rich media (Hm) or starved of iron and heme (DIP). Determination of gene expression in the TO16 mutant strain versus the ATCC 33277 wild-type strain showed changes in significantly lower number of genes and less significant changes in their expression levels compared with the TO6 mutant strain versus the A7436 wild-type strain, suggesting a smaller impact of PgFur on global gene expression in the ATCC 33277 strain. Therefore, we selected genes encoding potential virulence factors whose expression has been significantly changed in both *P. gingivalis* strains as examined by microarray analysis and determined their expression at various growth stages using RT-qPCR.

Among a variety of genes engaged in the regulation of virulence factors’ expression are those encoding potential transcription factors. We observed lower expression of *PGA7_00015190*/*PGN_1569* and *PGA7_00004090*/*PGN_0537* genes, especially in the early stationary growth phase in both TO6 and TO16 mutant strains (Additional file 3: Table S2). Both genes encode putative transcription factors belonging to the Crp/Fnr superfamily. Surprisingly, the expression pattern of the *PGA7_00009810*/*PGN_0970* gene encoding sigma factor differed in TO6 and TO16 mutant strains compared with the wild-type strains. Also worth noting are differences in expression of genes encoding proteins associated with the CRISPR/Cas system. Both TO6 and TO16 mutant strains demonstrated lower expression of these genes compared with the wild-type counterparts, especially during growth under high iron/heme conditions (Additional file 3: Table S2). We hypothesize that the above-mentioned proteins would participate in a multilayer regulatory network in collaboration with PgFur and also with other, so far unidentified proteins.

### Proteolytic activity of *P. gingivalis*

**Fig. 5:**
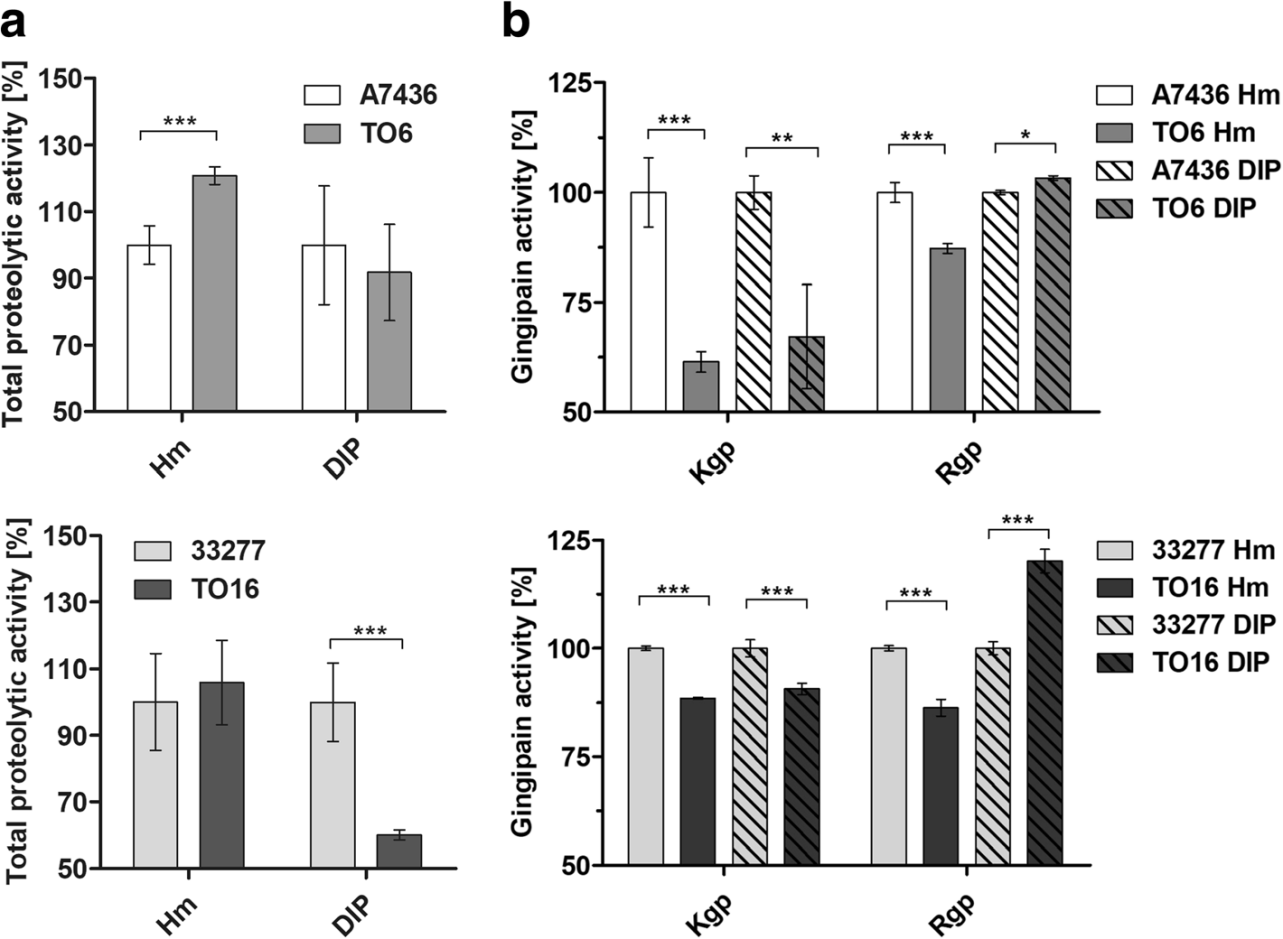
Total proteolytic (**a**) and gingipain activities (**b**) of *P. gingivalis* wild-type (A7436 and ATCC 33277) and *pgfur* mutant (TO6 and TO16) strains. Bacteria were grown under high- (Hm) or low-iron/heme (DIP) conditions in liquid culture medium for 24 h and proteolytic activity of whole cultures against azocasein (**a**) or protease substrates specific for arginine- (Rgp) or lysine-specific (Kgp) gingipains was determined. *P < 0.05, **P < 0.01, ***P < 0.001

**Fig. 6:**
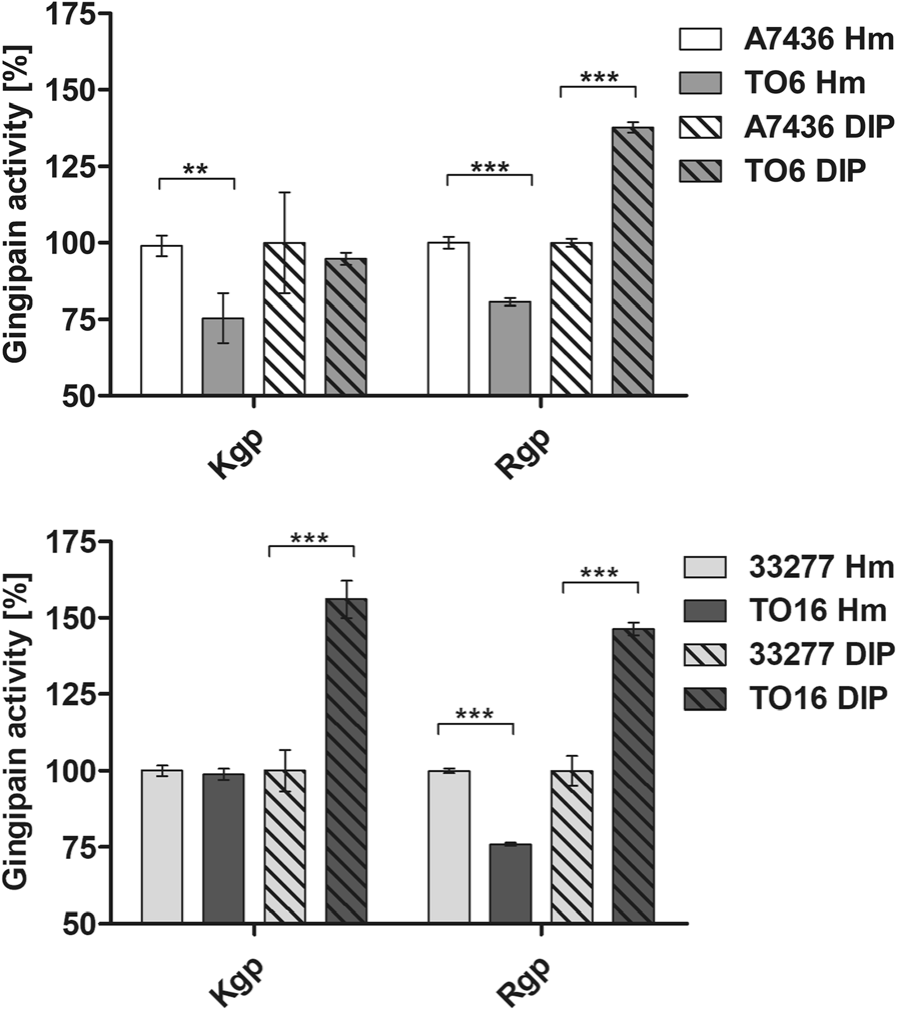
Gingipain activity of *P. gingivalis* wild-type (A7436 and ATCC 33277) and *pgfur* mutant (TO6 and TO16) strains. Bacteria were grown under high- (Hm) or low-iron/heme (DIP) conditions in liquid culture medium for 24 h and gingipain activity in separated culture medium was determined against protease substrates specific for arginine- (Rgp) or lysine-specific (Kgp) gingipains. *P < 0.05, **P < 0.01, ***P < 0.001

Recently, we demonstrated that PgFur could be involved in the regulation of hemolytic/proteolytic activity of *P. gingivalis* [31]. Therefore, in this study, we first determined the total proteolytic activity of whole *P. gingivalis* cultures using azocasein as a protease substrate. When bacteria were grown under high-iron/heme conditions (Hm), only the TO6 mutant strain exhibited significantly higher proteolytic activity compared with the A7436 wild-type strain (Fig. 5 a), corroborating data presented in Fig. 2c. Under low-iron/heme conditions (DIP), both TO6 and TO16 mutant strains exhibited lower proteolytic activity, but only the TO16 strain demonstrated statistically significant differences. Then, we determined gingipain activity of whole *P. gingivalis* cultures using substrates specific for Rgp and Kgp gingipains (Fig. 5 b). Both TO6 and TO16 mutant strains, with more pronounced differences observed in the case of the TO6 mutant strain, exhibited significantly lower Kgp activity under both high- (Hm) and low-iron/heme (DIP) conditions compared with the wild-type strains. Rgp activity was lower in both TO6 and TO16 mutant whole cultures under high-iron/heme (Hm) conditions. In contrast, under low-iron/heme (DIP) conditions Rgp activity in whole bacterial cultures was higher in the case of both mutant strains compared with the wild-type counterparts. Further, we determined the activity of soluble gingipains secreted into the culture media (Fig. 6). Under high-iron/heme (Hm) conditions, Kgp activity was significantly lower in the case of the TO6 mutant strain, whereas under low-iron/heme (DIP) conditions the TO16 mutant strain exhibited significantly higher Kgp activity. A similar pattern of Rgp activity was observed for both TO6 and TO16 mutant strains, being lower under high-iron/heme (Hm) conditions and higher under low-iron/heme (DIP) conditions. These data are in contrast to results obtained from RT-qPCR analysis (Additional file 3: Table S2), suggesting the potential involvement of PgFur in the regulation of processing of gingipains.

### Glycosylation of *P. gingivalis* macromolecules

**Fig. 7:**
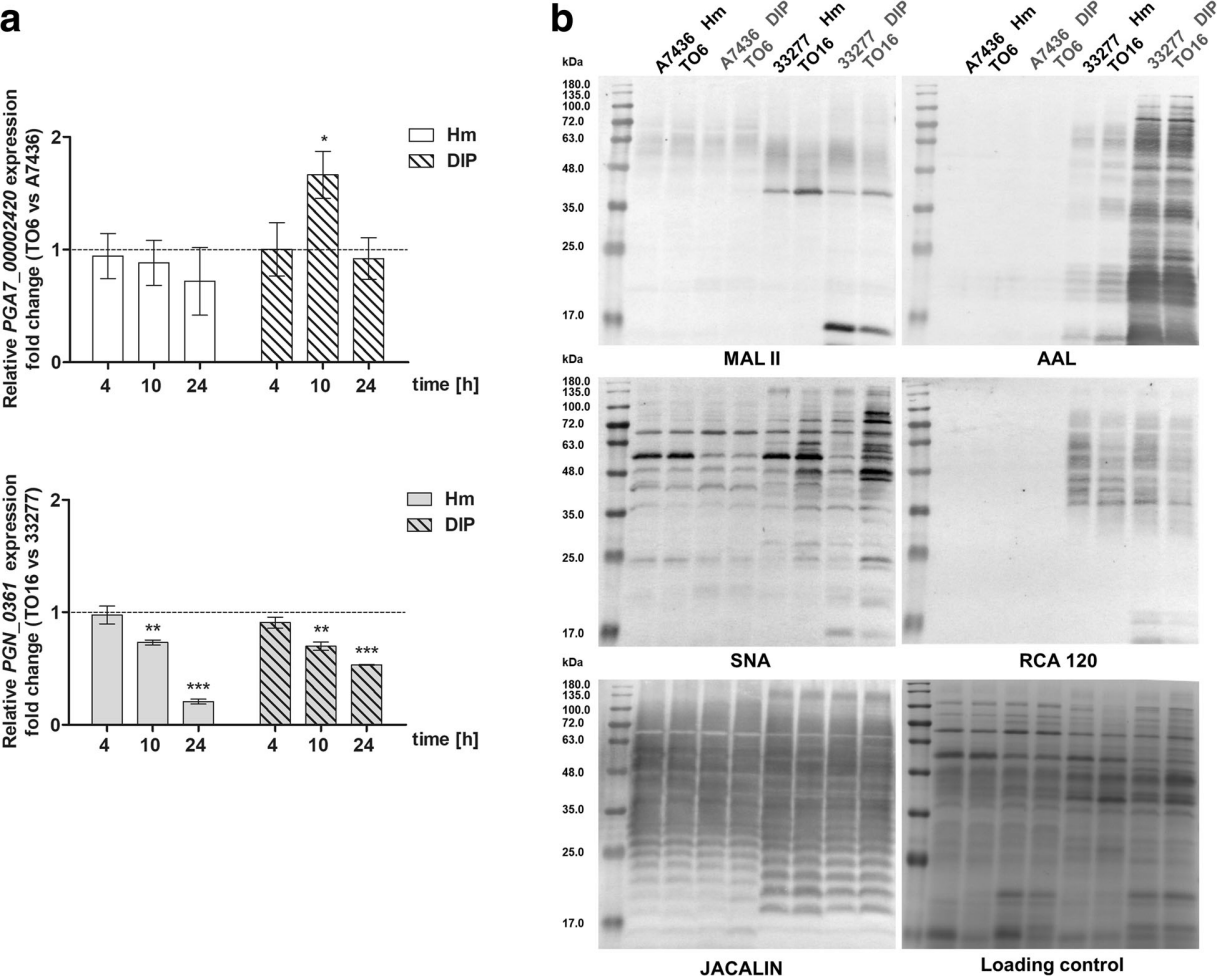
Analysis of expression of selected glycosyltransferase gene and glycosylation pattern of glycoconjugates produced by *P. gingivalis* wild-type (A7436 and ATCC 33277) and *pgfur* mutant (TO6 and TO16) strains. Bacteria were grown under high- (Hm) or low-iron/heme (DIP) conditions in liquid culture medium. **a** Relative gene expression was determined at the indicated time points in *pgfur* mutant (TO6 and TO16) strains versus wild-type (A7436 and ATCC 33277) strains by RT-qPCR. **b** Whole bacterial lysates were separated by SDS-PAGE, transferred onto nitrocellulose membranes and probed with selected lectins. MAL II, sialic acid; AAL, fructose linked to *N*-acetylglucosamine with α-1,6 linkage or to *N*-acetyllactosamine with α-1,3 linkage, arabinose; SNA, sialic acid linked to the terminal galactose with α-2,6 or α-2,3 linkage; RCA 120, galactose and *N*-acetylgalactosamine; JACALIN, galactose linked to *N*-acetylgalactosamine with β-1,3 linkage. Equal loading of proteins was visualized in SDS-PAGE gels by staining with Coomassie Brilliant Blue G-250 (loading control). *P < 0.05, **P < 0.01, ***P < 0.001

Microarray analysis suggested differences between the wild-type and mutant strains in the expression of some genes potentially involved in glycosylation processes. As shown in Fig. 7a, one of the selected glycosyltransferases examined was differentially expressed in the mutant strains compared with the wild-type strains, especially under low iron/heme conditions. To visualize potential changes in glycosylation of glycoconjugates produced by *P. gingivalis*, lectin blotting analysis was employed. As shown in Fig. 7 b, significant differences in glycosylation pattern could be observed between A7436 and ATCC 33277 wild-type strains (mostly visible in reactivity with MAL II, AAL, RCA 120, and JACALIN), as well as between growth conditions (mostly visible in reactivity with AAL and SNA). Moreover, we observed changes in glycosylation of glycoconjugates produced by the TO16 mutant strain compared with the ATCC 33277 wild-type strain (mostly visible in reactivity with MAL II, RCA 120 and SNA).

### Production of HmuY protein

**Fig. 8:**
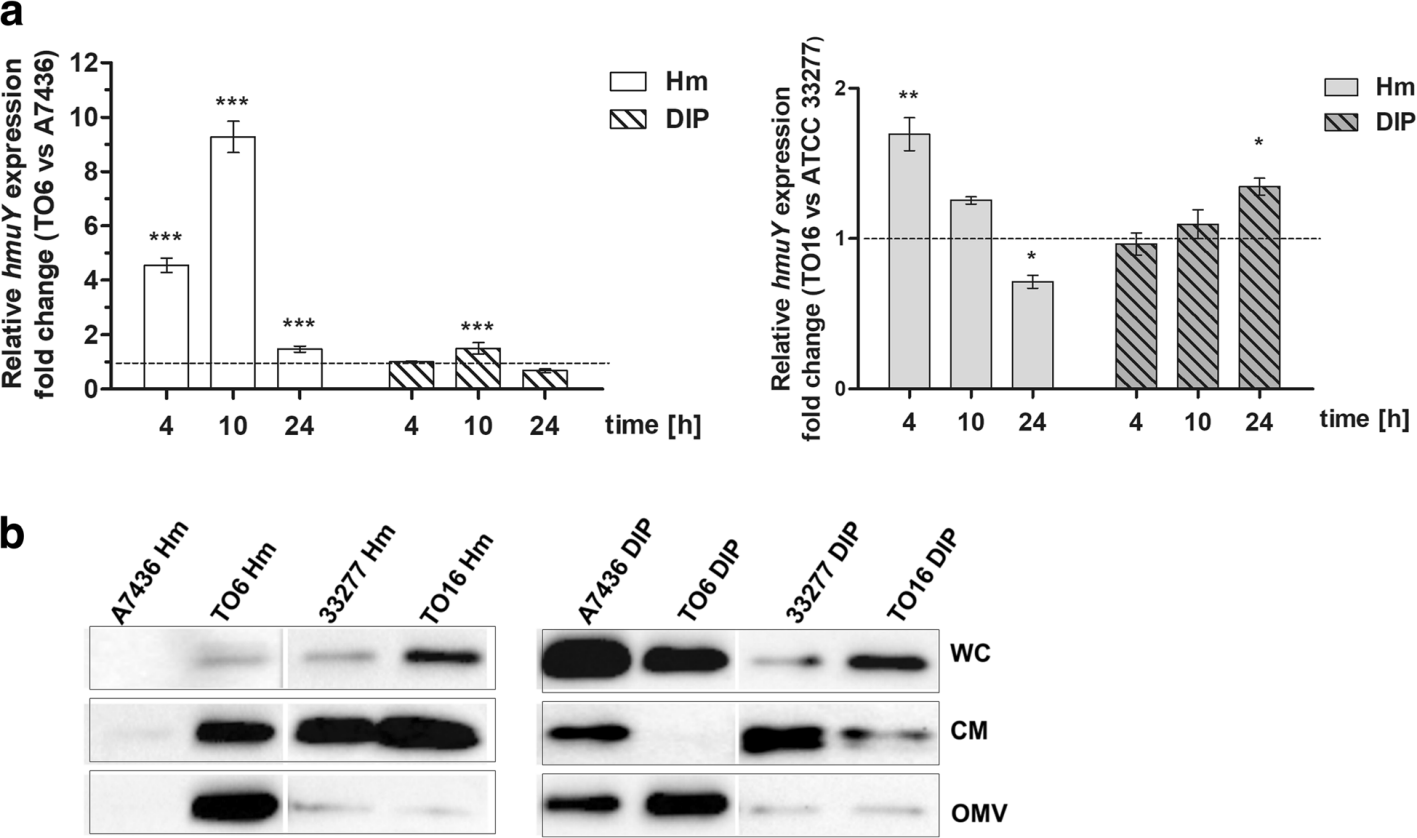
Expression of *P. gingivalis hmuY* gene. Determination of *hmuY* transcript was analyzed using RT-qPCR (**a**) and HmuY protein using Western blotting with anti-HmuY antibodies (**b**). Bacteria were grown under high- (Hm) or low-iron/heme (DIP) conditions in a liquid culture medium. Relative gene expression was determined in *pgfur* mutant (TO6 and TO16) strains versus wild-type (A7436 and ATCC 33277) strains. WC, whole bacterial culture; CM, culture medium; OMV, outer-membrane vesicles. *P < 0.05, **P < 0.01, ***P < 0.001

Recently, we demonstrated that the TO6 mutant strain produced higher levels of *hmuY* transcript [31]. Here we observed a similar tendency in the case of the TO16 mutant strain, although the differences were significantly less pronounced (Additional file 3: Table S2 and Fig. 8 a). In addition, a different pattern of mRNA encoding HmuY protein after inactivation of the *pgfur* gene could be observed depending on the growth phase (Additional file 3: Table S2 and Fig. 8 a). Inter-strain differences could also be observed in production of the HmuY protein (Fig. 8 b). In general, A7436 and TO6 strains produced higher levels of the HmuY protein, determined in whole bacterial cultures (WC), compared with ATCC 33277 and TO16 strains, all cultured under low-iron/heme conditions. Complementing the lack of the *pgfur* gene caused partial restoration of the ability of the mutant cells to produce HmuY protein, as determined in whole bacterial cultures (Additional file 2: Figure S1). Under high-iron/heme conditions (Hm), both mutant strains produced larger amounts of the HmuY proteins compared with their wild-type counterparts (Fig. 8 b). Under iron and heme starvation (DIP), a similar pattern was observed for ATCC 33277 and TO16 strains. Analysis of OMVs demonstrated a similar expression pattern of the HmuY protein compared with the whole cells (WC). When analyzing soluble HmuY protein shed from the bacterial cells and OMVs and accumulated in the culture medium (CM), the main difference was visible in the HmuY protein detected in the wild-type strains and mutant strains under iron/heme-limited conditions. TO6 and TO16 mutant strains secreted and accumulated in the culture medium lower amounts of soluble HmuY protein (Fig. 8 b).

### Infection ability of *P. gingivalis* strains against macrophages

**Fig. 9:**
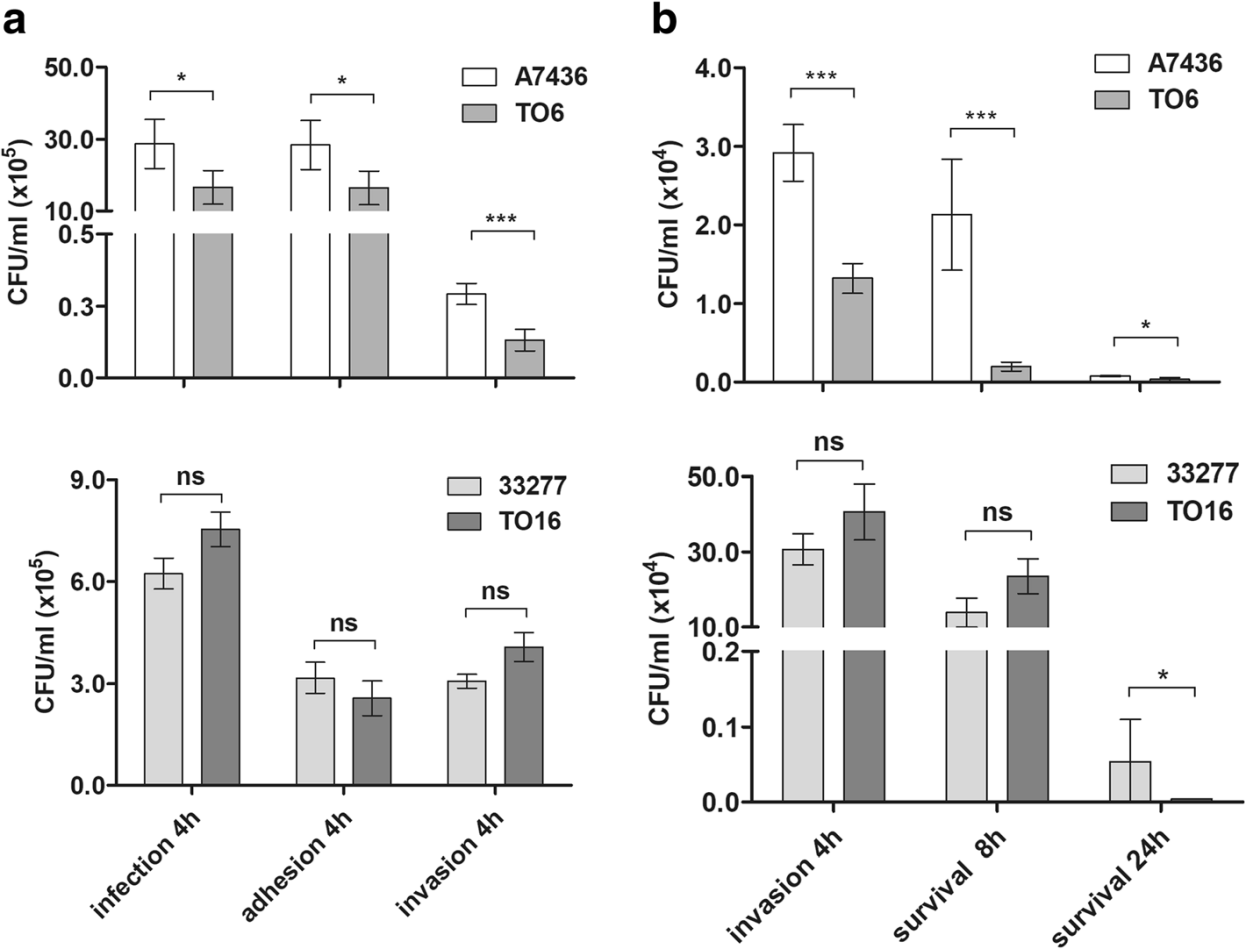
Infection ability of *P. gingivalis pgfur* mutant strains against human macrophages. **a** The infection efficiency was determined by counting live bacteria adhering to macrophages (adhesion) and live bacteria invading macrophages (invasion). **b** Survival ability was determined by counting live bacteria which invaded and survived inside macrophages. *P < 0.05, ***P < 0.001, ns – statistically not significant

To demonstrate the importance of PgFur for pathogenicity, we analyzed infection ability (adhesion and invasion) and intracellular survival ability of two sets of *P. gingivalis* wild-type and *pgfur* mutant strains after infection of THP-1-derived macrophages. In general, the most pronounced differences were observed between A7436 and TO6 strains compared with ATCC 33277 and TO16 strains (Fig. 9). In agreement with our previous study [31], *P. gingivalis* adhesion to the macrophages was lower for the TO6 mutant strain compared with the A7436 wild-type strain (Fig. 9). Surprisingly, no significant differences were observed for the TO16 mutant strain compared with the ATCC 33277 wild-type strain. Importantly, both mutant strains exhibited lower survival ability inside macrophages compared with the wild-type strains, confirming involvement of PgFur in the regulation of expression of genes required for efficient infection.

## Discussion

*P. gingivalis* expresses its transcriptome and proteome in response to environmental changes [19, 31, 38–40]. Cell density and iron/heme availability are important signals affecting the physiology of bacterial cells and are important for the pathogenicity of *P. gingivalis*. However, it has been suggested that some strains may have the capacity to cause dysbiosis and disease, and differences between strains in the ability to cause localized or systemic infections have been observed in animal model studies [41–47]. For example, the ATCC 33277 strain (non-encapsulated) forms higher biofilm structures, is highly fimbriated, but less virulent, whereas the A7436 strain (encapsulated) is less fimbriated, produces smaller biofilm structures, but exhibits higher virulence in animal model studies [48–52]. Several reports have shown that diversity in production of specific virulence factors by *P. gingivalis* might be caused by several mechanisms, including specific domain rearrangements and allelic exchange [43, 47, 51, 53–56]. One example is the two-component regulatory system composed of the histidine kinase HaeS and the response regulator HaeR. The HaeSR system plays a role in regulation of the *hmu* operon as well as of genes encoding gingipains [57]. The ATCC 33277 strain lacks HaeS kinase due to the partial deletion of the *haeS* gene. However, the introduction of this gene into the ATCC 33277 strain allowed observation of the restoration of this system present in the W83 strain, which is highly similar to the A7436 strain. This fact could partly explain the differences in gene expression of the *hmu* operon and the different amounts of the HmuY protein produced by A7436 and ATCC 33277 strains. This may also enable one to produce different phenotypes by *P. gingivalis* strains, especially those which are more useful for bacterial survival under hostile host conditions, allowing more efficient nutrient uptake and avoidance of the host response.

Recently, we [31] and others [58, 59] demonstrated that PgFur does not regulate iron homeostasis in a manner typical for classical Fur proteins by changes of expression of genes usually involved in this process in other bacteria. Our data [31] are in accordance with the results published by Anaya-Bergman et al. [58] and Butler et al. [59], although regulation of transcription depending on iron and heme binding to the protein and a role in heme-responsive biofilm development have been suggested for the *P. gingivalis* Fur homolog by Butler et al. [60]. We found that expression of other proteins involved in iron and heme acquisition, namely some hemagglutinins, hemolysins and proteases, which are the most important *P. gingivalis* virulence factors, could be regulated by PgFur [31 and this study]. Interestingly, among them are genes encoded by the *hmu* operon, allowing *P. gingivalis* to efficiently acquire heme [17]. As shown previously [31] and in the present study, inactivation of the *pgfur* gene caused changes in expression of *hmuY* gene during *P. gingivalis* growth. Production of higher *hmuY* transcript as well as HmuY protein levels during *P. gingivalis* cultivation under rich iron and heme conditions of the TO6 and TO16 mutant strains compared with the wild-type strains suggests that the *hmu* operon can be regulated by PgFur. It is worth noting at this stage of our studies that *hmuY* expression is probably not only regulated by a simple, direct interaction between PgFur and the *hmuY* promoter region. We assume that PgFur, as a member of a complex regulatory network, might regulate expression of other regulatory genes, including two-component systems and other transcriptional factors. It was shown before [61–64] that Fur protein may influence indirectly gene regulation through proteins from the Crp/Fnr superfamily. A decrease in expression of the two genes encoding putative transcriptional factors from the Crp/Fnr family in both TO6 and TO16 compared with the wild-type strains confirmed a similar relationship between PgFur and proteins encoded by *PGA7_00015190*/*PGN_1569* and *PGA7_00004090*/*PGN_0537* genes in *P. gingivalis*. However, further studies are required to determine the mutual dependence of these proteins.

In bacterial liquid media [18, 19] and inside host cells [16], mRNA encoding *hmuY* and HmuY protein are produced with higher yields under low iron/heme conditions. In order to efficiently bind and deliver heme back to the bacterial cell, HmuY is released from the lipid anchor by specific cleavage by Kgp gingipain [19, 21]. We observed smaller amounts of HmuY protein accumulated in the culture medium in the *pgfur* mutant strains, which might be, at least in part, explained by lower Kgp activity exhibited by cultures of TO6 and TO16 mutant strains. Based on our data, one could assume that such an effect might result from involvement of PgFur in the regulation of processes responsible for gingipain processing and maturation, including glycosylation. Several studies have demonstrated that glycosylation is one of the most important modifications used by *P. gingivalis* to increase its virulence. Bacterial glycosylation is related mainly to macromolecules exposed to the external environment [65] and is important for bacterial interactions with and adhesion to the host cells [66]. Among the best-characterized glycoconjugates are gingipains [67, 68], an outer membrane OMP85 protein [69], and Mfa1 fimbriae [70]. Differences in glycosylation observed between the wild-type strains examined in the present study could result from the differences in production of some glycosylated macromolecules in the ATCC 33277 strain [69–71]. It was shown before that changes in glycosylation of macromolecules may influence colony morphology of *P. gingivalis* and other bacteria [72–74]. In agreement with those findings, the TO16 mutant strain differed in glycosylation pattern compared with the ATCC 33277 wild-type strain. It can explain, at least in part, the difference in colony shape of the mutant TO16 strain. In addition, some subtle changes were demonstrated between the wild-type and *pgfur* mutant strains.

We demonstrated that during prolonged culture on a solid medium the TO6 mutant strain formed light-colored colonies, unlike A7436 and TO16 strains. Lack of pigmentation was reported for *P. gingivalis* strains with dysfunction of some proteins, including those of the type IX secretion system [75–77]. Disturbance in gingipain secretion and maturation may lead to lower ability of the bacteria to accumulate heme on the bacterial cell surface. In addition, this fact might explain the increased expression of the *kgp* gene at the transcriptional level, especially in the early growth phase of the *pgfur* mutant strains, despite the decrease in Kgp proteolytic activity. Moreover, we observed discrepancy between enzymatic activity of a total pool of Kgp or Rgp gingipains and their secreted, soluble forms. Similar effects were reported by others in case of *P. gingivalis* mutants with affected gingipain production (e.g., [78, 79]). Our results suggest that multicomponent maturation pathways of gingipains could be affected due to the lack of functional *pgfur* gene. Therefore, we suggest that PgFur can participate rather indirectly in a regulatory network responsible for gingipain production and activity. Moreover, we believe that the observed dissimilarity in both mutant strains compared with the wild-type strains might be explained, at least in part, by differences in glycosylation and encapsulation of the A7436 strain.

Our recent study [31] showed that the TO6 mutant strain differed in composition of membrane-associated proteins. Together with findings from the present study, including changes in glycosylation between *pgfur* mutant strains and respective wild-type strains as well as differences between A7436 and ATCC 33277 wild-type strains, we tested interactions between *P. gingivalis* and other periodontopathogens. We found that *pgfur* gene differently influences the interaction between both wild-type strains of *P. gingivalis* and *P. intermedia* or *T. forsythia.* This effect might be explained by the difference in composition of the outer membrane of ATCC 33277 and A7436 strains due to the encapsulation of the latter. It is also worth noting that A7436 and ATCC 33277 strains differ in fimbriae production, and the A7436 strain does not produce short Mfa1 fimbriae, composed of glycosylated proteins. This might also, at least in part, explain the higher ability of the ATCC 33277 strain to co-aggregate with *S. gordonii* compared with the A7436 strain. Our findings are in agreement with data published by Park et al. [80], who demonstrated the importance of Mfa1 fimbriae for synergistic biofilm formation between *P. gingivalis* and *S. gordonii*.

Macrophages belong to essential components of the host immune system involved in the pathogenesis of a variety of inflammatory diseases [81, 82]. Elevated numbers of macrophages with increased levels of pro-inflammatory cytokines, which significantly contribute to tissue breakdown, were found in samples derived from chronic periodontitis patients [82–84]. It has been demonstrated that *P. gingivalis* can persist in naïve (MØ) and M2-like macrophages, but not in M1-like macrophages for 24 h [85]. On the other hand, activated M1 macrophages were more prevalent and the M1/M2 ratio was found to be increased in gingival tissues derived from patients with chronic periodontitis [82]. Recently, we demonstrated that the *P. gingivalis* A7436 strain not producing PgFur protein exhibited significantly diminished ability to adhere to host cells, and most importantly to invade HeLa epithelial cells, as well as U937 and THP-1 macrophages, suggesting that this protein is crucial for efficient infection of host cells [31]. In the present study, we observed a difference in the impact of PgFur protein on the interaction of different *P. gingivalis* wild-type strains with macrophages. We believe that the observed differences in outer membrane composition may also be crucial for this process. A difference in production of fimbriae and glycoconjugates may influence the ability of *P. gingivalis* to adhere to and invade macrophages. Another explanation might be a decrease in expression of genes encoding components of the CRISPR/Cas system in *pgfur* mutant strains compared with the wild-type counterparts. Recently, Heidrich et al. [86] suggested a novel function of the CRISPR/Cas system of *Neisseria meningitidis* by demonstration of lower adhesion ability of a strain lacking the *cas9* gene. In accordance with those findings, differences observed for mutant strains could be explained by changes in the composition of CRISPR/Cas-associated proteins (e.g., disruption of gene encoding Cas1 protein in the ATCC 33277 strain) and their expression, which results in significant differences observed only in the A7436 strain. As mentioned above, also impairment in pigmentation of the TO6 mutant strain may explain its lower survival ability inside macrophages. Pigmentation was considered one of the major virulence factors of *P. gingivalis*, associated with colonization, maintaining a local anaerobic environment by binding oxygen molecules, and defense against reactive oxygen species produced by immune cells. Data obtained in this study confirmed that bacterial tolerance and the response to oxidative stress might be crucial for *P. gingivalis* survival inside host cells. Based on our results, one may conclude that inactivation of the *pgfur* gene in A7436 results in significantly lower efficiency to adhere to, invade and survive inside THP-1-derived macrophages. However, compared with the ATCC 33277 strain, the A7436 strain is able to survive more efficiently inside macrophages and subsequently spread between the cells, thus increasing its virulence potential.

## Conclusions

In this study, we present for the first time a comparative analysis of *pgfur* gene inactivation effects in less and more virulent *P. gingivalis* strains. We demonstrate that inactivation of the *pgfur* gene in the *P. gingivalis* ATCC 33277 strain is reflected in significantly less visible changes in expression of genes encoding potential virulence factors compared with the *P. gingivalis* A7436 strain. Based on our data, one may draw the general conclusion that the two strains demonstrate different phenotypes not only when in the wild-type form but importantly also upon *pgfur* gene inactivation.

## Additional files

Additional file 1: **Table S1.** Primers used in this study. (PDF 132 kb)

Additional file 2: **Figure S1.** Complementation of *pgfur* inactivation in *P. gingivalis* mutant strains. Bacteria were grown under high-iron/heme (BM + Hm) conditions in liquid culture medium for 24 h. Optical density of the bacterial cultures after 24 h (**a**) and HmuY protein production in whole cell cultures (**b**) were determined. A7436, ATCC 33277 – wild-type strains; TO6, TO16 – *pgfur* mutant strains; TO6 + *pgfur*, TO16 *+ pgfur*; complemented *pgfur* mutant strains. ***P < 0.001, ns – statistically not significant. (PDF 104 kb)

Additional file 3: **Table S2.** RT-qPCR analysis of expression of selected genes in *P. gingivalis pgfur* mutant strains (TO6 and TO16) versus wild-type strains (A7436 and ATCC 33277). (PDF 135 kb)

Additional file 4: **Figure S2.** Heme binding to whole *P. gingivalis* cells. (PDF 94 kb)

## Data Availability

Data generated and analyzed in this study are included in this published article and its supplementary information files or are available upon request.
