# Supplementary material for: PgFur participates differentially in expression of virulence factors in more virulent A7436 and less virulent ATCC 33277 Porphyromonas gingivalis strains
Source: BMC Microbiol. 2019 Jun 11;19:127. doi: 10.1186/s12866-019-1511-x (PMC6558696; doi:10.1186/s12866-019-1511-x)
Supplement: Supplementary file 1 — Table S1. Primers used in this study. (PDF 132 kb) [file 12866_2019_1511_MOESM1_ESM.pdf]

Additional file 1: Table S1 Primers used in this study.

Primer	5'→3' oligonucleotide sequence	Locus ID (gene abbreviation)	Description (reference)
F_fsr_mut<br>R_fsr_mut	[illegible]<br>[illegible]	FGA7_0001 4370 (*pgfsr*)	Amplify DNA fragment encompassing *pgfsr* gene with *ermF* insertion in the TO6 mutant strain, which enabled construction of the TO16 mutant strain (this study)
F_PgFsr_XbaI<br>R_PgFsr_BamHI	[illegible]<br>[illegible]	FGA7_0001 4370 (*pgfsr*)	Amplify fragment of *pgfsr* gene including promoter, used for cloning into pTIO-tetQ plasmid for complementation of the TO6 and TO16 mutant strains (this study)
rtPURAF<br>rtPURAR	[illegible]<br>[illegible]	FGA7_0001 4370, FGN_1305 (*pgfsr*)	Amplify fragment of gene used in qRT-PCR [31]
16SrRNA-F<br>16SrRNA-R	[illegible]<br>[illegible]	FGA7_00000960, FGN_r0001 (16S rRNA)	Amplify fragment of gene used in qRT-PCR [36]
F_FG0996<br>R_FG0996	[illegible]<br>[illegible]	FGA7_0001 5190, FGN_1369 (transcription factor gene, *crp/fnr*)	Amplify fragments of genes used in qRT-PCR (this study)
F_FG1373<br>R_FG1373	[illegible]<br>[illegible]	FGA7_00004090, FGN_2337 (transcription factor gene, *crp*)	
F_FG1882<br>R_FG1882	[illegible]<br>[illegible]	FGA7_00018490, FGN_1927 (*cas1*)	
F_FG1882_2<br>F_FG1882_2	[illegible]<br>[illegible]	FGN_1925 (*cas1*)	
F_FG1883<br>R_FG1883	[illegible]<br>[illegible]	FGA7_00018500, FGN_1928 (*cas6*)	
F_FG1884<br>R_FG1884	[illegible]<br>[illegible]	FGA7_00018510 (CRISPR-associated gene)	
F_FG1884_2<br>R_FG1884_2	[illegible]<br>[illegible]	FGN_1929 (CRISPR-associated gene)	
F_FG1885<br>R_FG1885	[illegible]<br>[illegible]	FGA7_00018520, FGN_1930 (*cas4*)	
F_FG1886<br>R_FG1886	[illegible]<br>[illegible]	FGA7_00018530 (*cas4*)	
F_FG1886<br>R_FG1886_2	[illegible]<br>[illegible]	FGN_1931 (*cas4*)	
F_FG1887<br>R_FG1887	[illegible]<br>[illegible]	FGA7_00018540, FGN_1932 (*cas1/Rkme2*)	
F_FG2020<br>R_FG2020	[illegible]<br>[illegible]	FGA7_00018680, FGN_1966 (*cas8*)	
F_FG0264<br>R_FG0264	[illegible]<br>[illegible]	FGA7_00002420, FGN_2061 (glycosyltransferase gene)	
F_FG0270<br>R_FG0270	[illegible]<br>[illegible]	FGA7_00002460, FGN_2068 (*ragA*)	
F_FG0506<br>R_FG0506	[illegible]<br>[illegible]	FGA7_00004250, FGN_1466 (*rgpA*)	
F_FG0558<br>R_FG0558	[illegible]<br>[illegible]	FGA7_0001 8810, FGN_1416 (protease gene)	
F_FG0669<br>R_FG0669	[illegible]<br>[illegible]	FGA7_00012780, FGN_0705 (*fimA*)	
F_FG0719<br>R_FG0719	[illegible]<br>[illegible]	FGA7_00012310, FGN_0752 (*fimA*)	
F_FG0883<br>R_FG0883	[illegible]<br>[illegible]	FGA7_00009810, FGN_0970 (sigma factor gene)	
F_FG1049<br>R_FG1049	[illegible]<br>[illegible]	FGA7_00009230, FGN_1309 (*fimB*)	
F_FG1431<br>R_FG1431	[illegible]<br>[illegible]	FGA7_00007240, FGN_0903 (*fimR*)	
F_FG2024<br>R_FG2024	[illegible]<br>[illegible]	FGA7_00018890, FGN_1970 (*rgpA*)	
F_FG1844<br>R_FG1844	[illegible]<br>[illegible]	FGA7_00017110, FGN_1728 (*kgp*)	
HYqd_F<br>HYqd_R	[illegible]<br>[illegible]	FGA7_00004270, FGN_2358 (*hmuY*)	Amplify fragment of *hmuY* gene used in qRT-PCR [36]
F_hmuR<br>R_hmuR	[illegible]<br>[illegible]	FGA7_00004260, FGN_2357 (*hmuR*)	Amplify fragments of other genes composing *hmu* operon used in qRT-PCR (this study)
F_FG1353<br>R_FG1353	[illegible]<br>[illegible]	FGA7_00004250, FGN_2356	
F_FG1354<br>R_FG1354	[illegible]<br>[illegible]	FGA7_00004240, FGN_2355	
F_FG1355<br>R_FG1355	[illegible]<br>[illegible]	FGA7_00004230, FGN_2354	
F_FG1356<br>R_FG1356	[illegible]<br>[illegible]	FGA7_00004220, FGN_2353	
