# Supplementary material for: PgFur participates differentially in expression of virulence factors in more virulent A7436 and less virulent ATCC 33277 Porphyromonas gingivalis strains
Source: BMC Microbiol. 2019 Jun 11;19:127. doi: 10.1186/s12866-019-1511-x (PMC6558696; doi:10.1186/s12866-019-1511-x)
Supplement: Supplementary file 2 — Figure S1. Complementation of pgfur inactivation in P. gingivalis mutant strains. Bacteria were grown under high-iron/heme (BM + Hm) conditions in liquid culture medium for 24 h. Optical density of the bacterial cultures after 24 h (a) and HmuY protein production in whole cell cultures (b) were determined. A7436, ATCC 33277 – wild-type strains; TO6, TO16 – pgfur mutant strains; TO6 + pgfur, TO16 + pgfur; complemented pgfur mutant strains. ***P < 0.001, ns – statistically not significant. (PDF 104 kb) [file 12866_2019_1511_MOESM2_ESM.pdf]

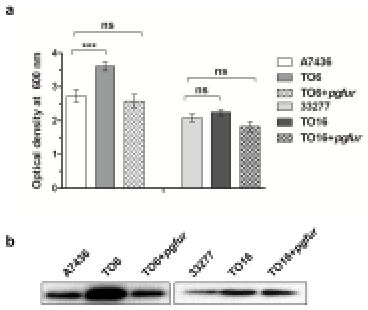

Additional file 2: Figure S1. Complementation of *pgfur* inactivation in *P. gingivalis* mutant strains. Bacteria were grown under high-iron/heme (BM+Hm) conditions in liquid culture medium for 24 h. Optical density of the bacterial cultures after 24 h (**a**) and HmuY protein production in whole cell cultures (**b**) were determined. A7436, ATCC 33277 – wild-type strains; TO6, TO16 – *pgfur* mutant strains; TO6+*pgfur*, TO16+*pgfur*; complemented *pgfur* mutant strains. ****P* < 0.001, ns – statistically not significant.
