# Supplementary material for: PgFur participates differentially in expression of virulence factors in more virulent A7436 and less virulent ATCC 33277 Porphyromonas gingivalis strains
Source: BMC Microbiol. 2019 Jun 11;19:127. doi: 10.1186/s12866-019-1511-x (PMC6558696; doi:10.1186/s12866-019-1511-x)
Supplement: Supplementary file 3 — Table S2. RT-qPCR analysis of expression of selected genes in P. gingivalis pgfur mutant strains (TO6 and TO16) versus wild-type strains (A7436 and ATCC 33277). (PDF 135 kb) [file 12866_2019_1511_MOESM3_ESM.pdf]

Additional file 3: Table S2 RT-qPCR analysis of expression of selected genes in *P. gingivalis pgfur* mutant strains (TO6 and TO16) versus wild-type strains (A7436 and ATCC 33277).

Class of encoded proteins	TO6 versus A7436							TO16 versus ATCC 33277						
	Gene	Hm			DIP			Gene	Hm			DIP		
		4 h	10 h	24 h	4 h	10 h	24 h		4 h	10 h	24 h	4 h	10 h	24 h
Proteins engaged in iron and heme uptake – *hmu* operon components	*hmuY*	4.75***	12.94***	1.76***	-1.07	1.30***	-1.21	*hmuY*	1.66**	1.16	-1.28*	1.25	1.12	1.29*
	hmuR	3.79***	14.59***	8.19***	-1.06	1.01	-1.33*	*hmuR*	1.56**	1.10	-1.33*	1.23	-1.36	1.92**
	PGA7_00004230	3.27***	13.77***	15.29***	-1.04	-1.1	-1.35*	*PGN_0556*	1.57**	1.12	-1.02	1.27	-1.54	2.00**
	PGA7_00004240	4.51***	20.36***	1.49*	1.17	1.13	-1.2	*PGN_0555*	1.64**	1.15	-1.00	1.33	-1.22	2.59**
	PGA7_00004230	2.72***	4.60***	-1.09	1.03	1.08	-1.24*	*PGN_0554*	1.25*	-1.23	-4.25***	1.06	-1.36	1.20
	PGA7_00004220	-1.41*	-1.12	1.58**	1.38*	1.85**	1.64**	*PGN_0553*	-1.02	-1.43	-1.68*	1.51*	1.10	1.16
Proteins engaged in iron and heme uptake	*feoB*	-1.61*	2.05**	-1.47*	1.16	1.30*	-1.04	*feoB*	-1.68**	-1.78*	-1.90***	-1.08	-1.50**	-1.35*
	fetB	-1.11*	1.03	-1.17	-1.09	1.75**	1.22	*fetB*	-1.32*	-1.26	-1.97***	-1.13	-1.31*	-1.85***
Proteases	*rgpB*	-1.34*	1.07	1.08	-1.66**	1.31*	-1.26*	*rgpB*	-1.29*	-1.36*	-2.11***	1.13	-1.01	-1.15
	kgp	1.73**	1.85**	1.97***	1.30	2.49***	1.47**	*kgp*	1.00	1.12	-2.85***	1.34*	-1.34**	-1.40*
	rgpA	-1.19	1.07	1.22*	-1.16	-1.19	-1.32*	*rgpA*	-1.62***	-2.23***	-2.91***	-1.62***	-2.53**	-1.66**
	PGA7_00013810	-1.52**	1.06	-1.92*	-1.38*	-1.35*	-1.18	*PGN_1416*	-1.95***	-2.78***	-3.27***	-1.20*	-3.43***	-1.91***
CRISPR associated proteins	-	-	-	-	-	-	-	*cas1*	-1.48**	-1.09	-1.74***	-1.01	-1.26*	1.01
	cas1	-1.14	-2.17**	-2.14***	1.14	1.20	-1.02	*cas1*	-1.50**	-1.34	-3.65***	2.05***	1.18	-1.63**
	cmr6	-1.15	-1.45**	1.13	-1.17	-1.18	-1.01	*cmr6*	-1.56**	1.19	-2.01*	1.42*	-1.22	1.13
	PGA7_00018310	-1.11	-1.39*	-2.00***	-1.50**	-1.16	-1.01	*PGN_1929*	-1.25*	1.24*	-1.84***	1.29	-1.69***	1.06
	cmr4	-1.05	-1.22	-1.44**	-1.19	-1.03	-1.08	*cmr4*	-1.42*	1.06	-1.70**	1.39*	-1.26	1.11
	cmr3	-1.12	-1.37*	-1.69**	-1.12	-1.00	1.26*	*cmr3*	ND	ND	ND	ND	ND	ND
	cas10/cmr2	-1.17	-1.14	-1.34*	-1.14	-1.18	1.18	*cas10/cmr2*	-2.00***	1.18	-1.61**	1.46*	-1.27	1.35*
	cas6	-1.27*	-1.90***	-1.42*	-1.28*	-1.21*	-1.13	*cas6*	-1.49**	1.20	-1.11	1.36**	-1.01	-1.05
Transcription regulators	*oxyR*	-1.35*	-1.15	-1.05	-1.00	2.26***	-1.17*	*oxyR*	1.17	1.19	-1.99***	1.22*	-1.03	1.19
	PGA7_00013190 (crp/fur)	-1.09	-1.22	-1.86*	-1.00	-1.06	-1.49**	*PGN_1569 (crp/fur)*	-1.12	-1.06	-1.92***	-1.12	-1.21*	-1.04
	hasS	-1.18	-1.20	-1.29*	-1.19	-1.05	-1.02	-	-	-	-	-	-	-
	PGA7_00009810	1.58**	3.15***	1.94***	2.33***	1.63*	1.57*	*PGN_0970*	-1.64**	-1.54**	-1.21	1.40*	-2.00**	1.11
	PGA7_00004090 (crp)	-1.22*	-1.46**	-1.81***	1.01	1.16*	-1.63**	*PGN_0557(crp)*	-1.25*	-1.11	-1.63***	-1.11	-1.00	-1.25**
	hasR	-1.33*	1.15	-1.34**	-1.08	-1.22*	-1.29*	*hasR*	-1.42*	-1.65**	-1.68**	1.20	-1.20*	-1.19

The data are shown as a relative fold change. Hm, iron/heme rich conditions; DIP, iron/heme limited conditions. ND, not detected. *p<0.05; **p<0.01; ***p<0.001.
