# Supplementary figures and images for: PgFur participates differentially in expression of virulence factors in more virulent A7436 and less virulent ATCC 33277 Porphyromonas gingivalis strains
Source: BMC Microbiol. 2019 Jun 11;19:127. doi: 10.1186/s12866-019-1511-x (PMC6558696; doi:10.1186/s12866-019-1511-x)

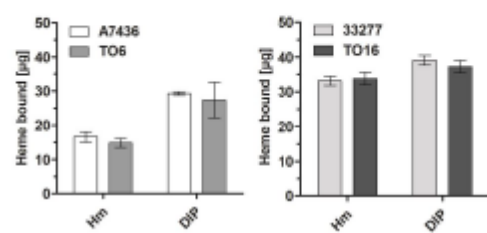

**Additional file 4:** Figure S2 Heme binding to whole *P. gingivalis* cells.

Supplement: Supplementary file 4 — Figure S2. Heme binding to whole P. gingivalis cells. (PDF 94 kb) [file 12866_2019_1511_MOESM4_ESM.pdf]
